# Predictive Factors of Mortality in Acute Aortic Dissection and Validity of the EuroSCORE Algorithm in a Small-Sized Cardiac Surgery Institution

**DOI:** 10.21470/1678-9741-2020-0053

**Published:** 2020

**Authors:** Facundo Rios, Diego Perez, Gerardo Soca, Ricardo Robaina, Victor Dayan

**Affiliations:** 1 National Institute of Cardiac Surgery, Montevideo, Uruguay.

**Keywords:** ROC Curve, Aneurysm, Dissecting, Postoperative Period, Coronary Artery Bypass

## Abstract

**Introduction:**

Acute aortic dissection (AAD) is a devastating surgical emergency, with high operative mortality. Several scoring algorithms have been used to establish the expected mortality in these patients. Our objective was to define the predictive factors for mortality in our center and to validate the EuroSCORE and Penn classification system.

**Methods:**

Patients who underwent surgery for AAD from 2006 to 2016 were retrieved from the institution’s database. Preoperative, operative and postoperative variables were collected. Observed and expected mortality was calculated by EuroSCORE. Logistic regression analysis and Cox regression analysis were performed to find predictors of operative mortality and survival, respectively. The receiver operating characteristic (ROC) curves were plotted for logistic EuroSCORE, and the area under the ROC curve (AUC) was calculated.

**Results:**

87 patients (27.6% female) underwent surgery for AAD. The mean age was 58.6±9.7 years. Expected and observed operative mortality was 25.8±15.1% and 20.7%, respectively. Penn Aa, Ab and Abc shared similar observed/expected (O/E) mortality ratio. The only independent predictor of operative mortality (OR: 3.63; 95% CI: 1.19-11.09) and survival (HR: 2.6; 95% CI: 1.5-4.8) was female gender. EuroSCORE showed a very poor prediction capacity, with an AUC=0.566.

**Conclusion:**

Female gender was the only independent predictor of operative mortality and survival in our institution. EuroSCORE is a poor scoring algorithm to predict mortality in AAD, but with consistent results for Penn Aa, Ab and Abc.

**Table:** 

Abbreviations, acronyms & symbols	
AAD	= Acute aortic dissection
ACP	= Antegrade cerebral perfusion
AMI	= Acute myocardial infarction
AUC	= Area under the curve
CI	= Confidence interval
CPB	= Cardiopulmonary bypass
HR	= Hazard ratio
IRAD	= International Registry of Acute Aortic Dissection
IRB	= Institutional Review Board
OR	= Odds ratio
ROC	= Receiver operating characteristic
STS	= Society of Thoracic Surgeons
TIA	= Transient ischemic attack

## INTRODUCTION

Acute aortic dissection (AAD) is a surgical emergency. Despite quick diagnosis and surgery, it has a high operative mortality, which ranges between 19% and 32%^[1,2]^. The explanation is mainly driven by its associated complications, such as cardiac tamponade, acute myocardial infarction (AMI), stroke and malperfusion syndrome.

Several authors have searched for predictors of mortality in patients with AAD. The Penn classification groups patients according to the presence of localized or general ischemia, among other things^[3]^. Scant data exists regarding the use of current scoring algorithms in small-sized cardiac surgery institutions.

Our aim was to evaluate predictors of mortality in patients with AAD and to try to validate the Penn classification and EuroSCORE in our center.

## METHODS

The Institutional Review Board (IRB) accepted the study and patient informed consent was waived. Data from the institution’s database was searched from 2006 until 2016 to identify patients who underwent surgery for AAD. No exclusion criteria was used. Baseline, operative and postoperative variables were extracted.

Cardiovascular risk factors, clinical characteristics and physical examination at the time of presentation were identified. Expected mortality was assessed according to EuroSCORE I, which is the only validated score in our country. Additionally, we calculated the time from the onset of pain until surgery. Patients were classified according to the Penn classification: Aa - absence of vessel malperfusion or circulatory collapse; Ab - branch vessel malperfusion with ischemia; Ac - circulatory collapse with or without cardiac involvement; Abc - branch vessel malperfusion and circulatory collapse^[3]^.

Operative mortality was defined as mortality within 30 days of surgery or during initial hospital admission.

### Surgical Technique

Midline sternal approach was used in all patients. Arterial cannulation was performed through the femoral, axillary artery or ascending aorta. Temperature during circulatory arrest varied according to the use of antegrade cerebral perfusion (ACP). Deep hypothermic (18°C) circulatory arrest was performed in cases in which no ACP was used and moderate hypothermia in cases in which it was used (22-28°C). A composite conduit (St Jude Medical) was used in cases in which dissection or aneurysmal dilatation involved the aortic root. In these cases, we performed a Bentall-De Bono procedure with coronary ostial reattachment. If only the ascending aorta were involved, we would replace the affected segment with a prosthetic conduit. The aortic valve was resuspended with 3 pledgeted non-absorbable sutures in each of the commissures or replaced in cases in which regurgitation was more than moderate. The aortic arch was explored in all cases during circulatory arrest to evaluate the presence of additional tears.

### Statistics

Continuous variables were expressed as mean±SD. Categorical variables were expressed as absolute value and percentages. Student’s t-test or Mann-Whitney test was used to compare continuous variables. Categorical variables were compared using chi-square test. Survival was analyzed with Kaplan-Meier curves and compared with log-rank test. The receiver operating characteristic (ROC) curves were plotted for the logistic EuroSCORE, and the area under the ROC curve (AUC) was calculated as an index for the predictive value of the model.

## RESULTS

From 2006 to 2016, 87 patients with AAD were operated on at our center. Mean age was 58.6±9.7 years and 27.6% were female (Table 1). Bicuspid aortic valve was present in 10.3% of patients.

**Table 1:** Baseline characteristics of the included patients.

Variable	
Age	58.6 (9.7)
Female	24 (27.6)
Hypertension	67 (77.9)
Diabetes	6 (7.1)
Tobacco use	33 (37.9)
Previous MI	4 (4.6)
Previous cardiac surgery	4 (4.6)
Stroke	3 (3.4)
PVD	1 (1.1)
Creatininemia	1.06 (0.55)
LVEF (%)	58.9 (7.7)
Bicuspid aortic valve	9 (10.3)
EuroSCORE I	25.8 (15.1)

LVEF=left ventricular ejection fraction; MI=myocardial infarction; PVD=peripheral vascular disease

Regarding the clinical presentation, 71.3% complained of chest pain and 12.6% of dorsal pain. Complications at the time of clinical evaluation were present in 27% of patients (Table 2). Of these, the most frequent was distal ischemia. Considering the time elapsed from initial pain onset to surgery, 21.8% of the patients underwent surgery in <24h, 56.3% between 24-48h and 10.3% between 48-72h.

**Table 2:** Clinical presentation of patients with acute aortic dissection (n=87).

Variable	
Pain localization	
Anterior chest	62 (71.3)
Dorsal	11 (12.6)
Complications	
Tamponade	3 (3.4)
Distal ischemia	5 (5.4)
Shock	3 (3.4)
Stroke	1 (1.1)
Lower limb ischemia	1 (1.1)
AMI	2 (2.3)
More than 1 complication	10 (10.3)
Pain onset to surgery	
<24h	19 (21.8)
24-48h	49 (56.3)
48-72h	9 (10.3)
>72h	10 (11.4)
Penn classification	
Aa	63 (72.4)
Ab	4 (4.6)
Ac	15 (17.2)
Abc	5 (5.7)

AMI=acute myocardial infarction

Patients who underwent surgery <24h had a higher incidence of complications (52.6% *vs*. 29.4%, *P*=0.06) and cardiac tamponade (26.3% *vs*. 5.9%, *P*=0.01). The mean time from symptom onset to surgery in patients with complicated AAD was 20.8h±19.6h, significantly lower than in patients without complications (34.1h±32.7h, *P*=0.02).

Penn Aa was the most common presentation (72.4%).

The most frequent surgical procedure performed was isolated replacement of the ascending aorta (78.2%) (Table 3). The aortic valve was replaced in 19 patients (21.8%). The mean duration of cardiopulmonary bypass (CPB) was 173±52 minutes and circulatory arrest was performed in 55.2% of cases, with a mean duration of 35±11 minutes. Cannulation of the femoral artery was the most frequent approach (49%) and the lowest temperature obtained was 18.7±7.1°C.

**Table 3:** Intraoperative characteristics of patients with acute aortic dissection.

Variables	
AA replacement	67 (78.2)
AA + valve replacement	15 (17.2)
Composite valve conduit	4 (4.6)
CPB time	173 (52)
Circulatory arrest (%)	55.2 (63.2)
Femoral cannulation	49 (56.3)
Axillary cannulation	14 (16.1)
Aortic cannulation	24 (27.5)
AXC time (min)	92 (32)
Circulatory arrest time	35 (11)
Lowest temperature	18.7 (7.1)
Hct pre-CPB (%)	35.7 (8.6)
Hct during CPB (%)	24.1 (4.9)
Hct post-CPB (%)	26.1 (5.5)

AA=ascending aorta; AXC=aortic cross-clamp; CPB=cardiopulmonary bypass

Expected operative mortality according to EuroSCORE was 25.8±15.1% and the observed mortality was 20.7%. Operative mortality was higher in female patients (37.5% *vs*. 14.3%, *P*=0.014) and in patients who underwent surgery <24h (21.1% *vs*. 12.1%, *P*=0.049). Operative mortality was similar in patients who required aortic valve replacement (10.5%) and those who did not AMI=acute myocardial infarction (23.5%, *P*=0.216). Observed/expected mortality was quite similar and constant in all Penn types, except for Penn Ac (Table 4).

**Table 4:** EuroSCORE and observed operative mortality in patients according to the Penn classification.

	Penn Aa	Penn Ab	Penn Ac	Penn Abc
EuroSCORE (SD)	25.7 (14.2)	37.2 (23.1)	22.7 (14.7)	26.4 (21.2)
Operative mortality (%)	11 (17.5)	1 (25)	5 (33.3)	1 (20)
O/E	0.68	0.67	1.45	0.75

The most frequent complication was transient ischemic attack (TIA) (18.4%). Mechanical assisted ventilation and ICU stay were 59.6±130.7 minutes and 6.3±12.1 days, respectively (Table 5).

**Table 5:** Postoperative outcomes in patients with acute aortic dissection.

Variables	
Total bleeding (ml)	1604 (1610)
Days in ICU	6.3 (12.1)
Mechanical ventilation (h)	59.6 (130.7)
Days to discharge	13.2 (13.7)
ARF	39 (44.8)
Dialysis	9 (10.3)
Stroke	10 (11.5)
TIA	16 (18.4)
Operative mortality (%)	18 (20.7)

ARF=acute renal failure; TIA=transient ischemic attack

Univariate regression analysis found only female gender (OR: 3.6; 95% CI: 1.2-10.7) and the lowest hematocrit in CPB (OR: 0.89; 95% CI: 0.80-0.99) as predictors of operative mortality. Circulatory arrest (OR: 3.6; 95% CI: 0.9-13.7) and surgery within 24 hours of symptom onset (OR: 3.0; 95% CI: 9.43-0.97), respectively, were at the borderline of statistical significance.

Comparison of baseline and operative variables between female and male patients showed that female patients had significantly lower hematocrit in CPB (21.9±4.6%) than male patients (24.9±4.8%, *P*=0.014). After multivariate regression analysis, only female gender resulted as an independent predictor for operative mortality (OR: 3.63; 95% CI: 1.19-11.09).

**Fig. 1:**
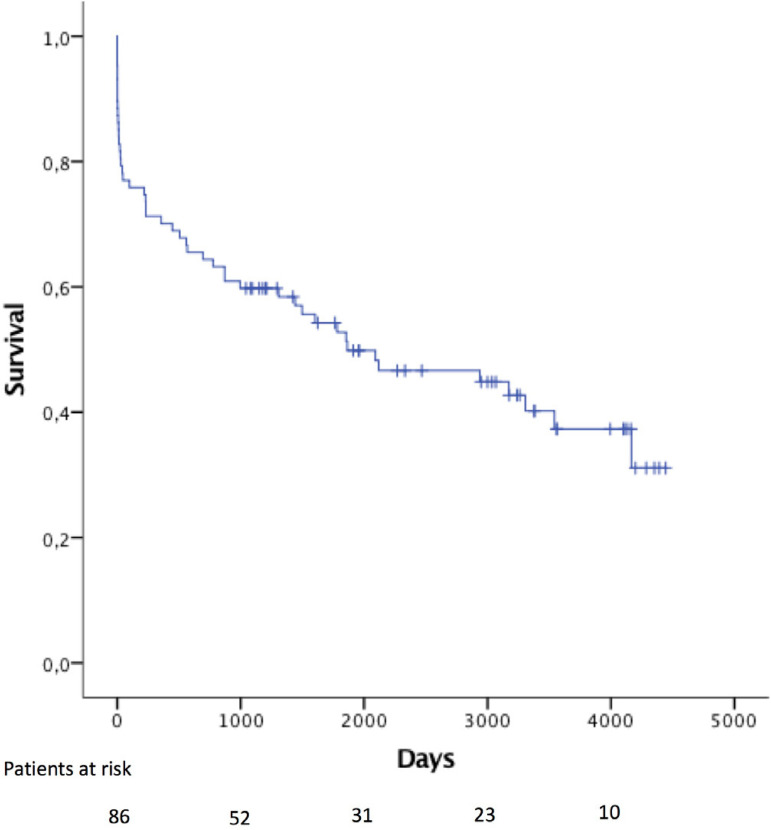
Overall survival in patients with AAD.

**Fig. 2:**
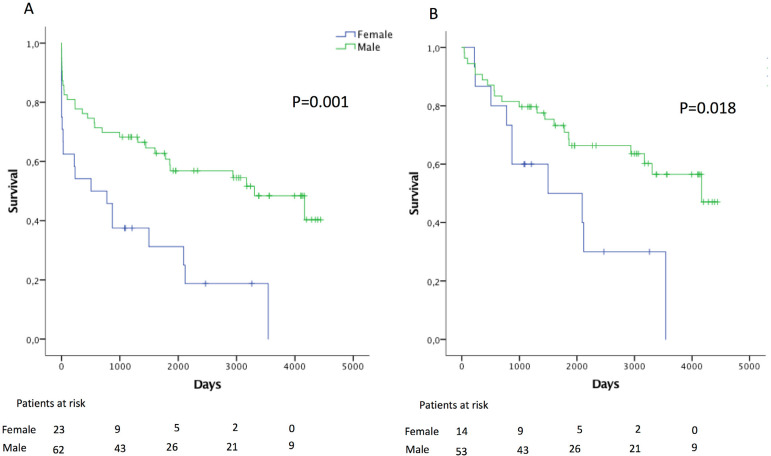
Survival according to gender before (A) and after (B) excluding operative mortality.

Five- and ten-year survival were 52.8±5.5% and 37.3±6.2%, respectively (Figure 1). Female patients had significant worse survival (HR: 2.6; 95% CI: 1.5-4.8), even after excluding operative mortality (HR: 2.4; 95% CI: 1.1-5.3) (Figure 2).

**Fig. 3:**
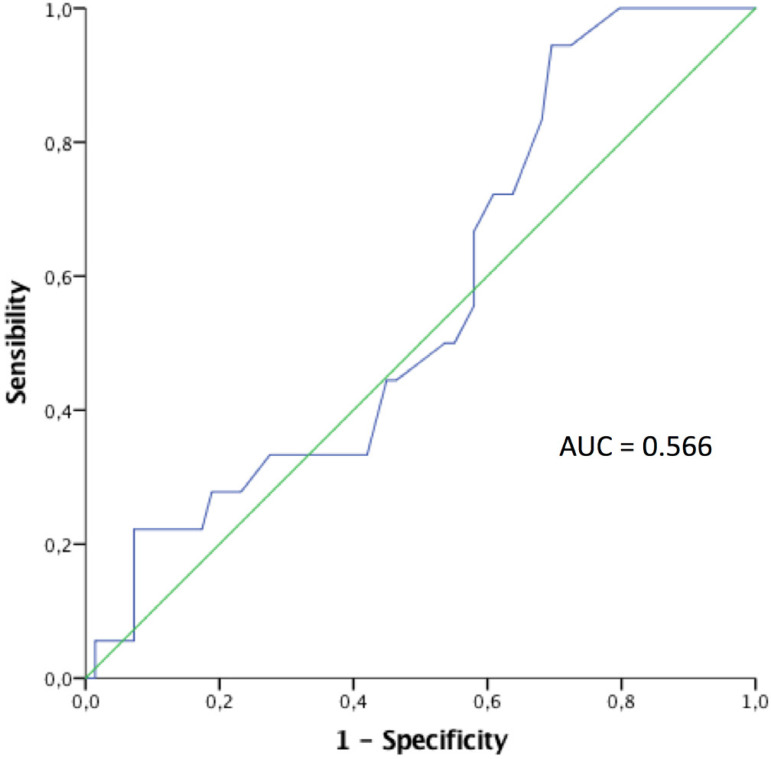
ROC analysis and AUC determination for EuroSCORE in operative mortality.

Regarding the predictive value of the EuroSCORE I (logistic) algorithm, the ROC showed an AUC of 0.566 (Figure 3).

## DISCUSSION

We found that AAD is associated with high operative mortality and low survival, similarly to other published reports^[2]^. We were able to show that the observed mortality was lower than the expected mortality according to EuroSCORE. Furthermore, the only predictor of mortality and long-term survival was female gender. EuroSCORE was not a useful algorithm to predict mortality, especially in patients with Penn Ac classification.

The International Registry of Acute Aortic Dissection (IRAD) has previously shown that female patients have increased mortality^[4]^. This difference in mortality between genders was seen especially in the 66-75-year-old group. In their study, Nienaber et al.^[4]^, reported 32% of operative mortality in female patients, which is similar to our data (37.5%). In contrast to these authors, we found no differences in time from pain onset and surgery, as well as the incidence in the complication rate between male and female patients. The main difference in our study was a lower hematocrit during CPB in female patients, which was related in the univariate analysis to mortality. This lower hematocrit could probably be explained by a lower body surface area in female patients and, therefore, greater hemodilution with the initiation of CPB. Similar to Gokalp et al.^[5]^, the arterial cannulation approach was not a predictor of mortality.

Patients operated on within 24 hours of symptom onset had a higher incidence of complications associated with AAD and cardiac tamponade, which could explain the higher mortality in this group. Delaying surgery after 24 hours may lead to exclude patients with serious complications who die before arriving to the hospital and, therefore, bringing into play natural selection^[6]^. The time from symptom onset to surgery in complicated and non-complicated patients in our series was lower than that reported by Trimarchi et al.^[7]^ (62.9 hours and 105.1 hours for unstable and stable patients, respectively). Similar to Trimarchi et al.^[7]^, patients with complicated AAD underwent surgery at a significantly shorter time-lapse from the symptom onset. It is classically accepted that untreated patients with AAD have an associated mortality rate of 1% to 2% per hour immediately after symptom onset^[8]^. We support this statement and promote surgery as quick as possible after diagnosis. We believe the cause of surgery delay in our series is mainly due to the difficulty in establishing the diagnosis of AAD due to the lower incidence of complications.

Penn type Aa was the most frequent presentation followed by Penn abc. Similar to Augoustides et al.^[3]^, mortality was lower in patients without any ischemic complications. When we related our observed mortality to the expected mortality, we found the O/E ratio was quite constant among Penn groups, except in Penn Ac. In patients with circulatory collapse (Penn Ac), EuroSCORE underestimated the expected mortality. This is probably since the score is not sensitive enough for this group of patients. The predictive value of EuroSCORE was quite low in our study group. The AUC was well below 0.75, which limits its role as a predictive algorithm for operative mortality in patients with AAD. Nishida et al.^[9,10]^ reported reliable prediction in 2006 using logistic EuroSCORE, but in 2014 found limited use of this scoring system. After the implementation of EuroSCORE II^[11]^, Howell et al.^[11]^ found that EuroSCORE II did not improve the prediction of mortality in high-risk patients. There is no defined data regarding the best risk score prediction model for AAD. Therefore, we believe that each center must evaluate the validity in AAD of its most used prediction algorithm and make use of all available tools in doing so.

### Limitations

The small number of patients inherent in our small center does not allow us to make further associations nor firmly conclude on our findings. Furthermore, it does not allow us to create our own scoring algorithm. The only validated score for cardiac surgery in our country is the EuroSCORE I, therefore, we do not have data regarding the EuroSCORE II or Society of Thoracic Surgeons (STS) score in the included patients to ascertain its validity in AAD.

## CONCLUSION

Female gender was the only predictor for operative mortality and long-term survival. Taking into consideration the Penn classification and the EuroSCORE, our data suggest that, although EuroSCORE is a poor algorithm to predict mortality, its prediction model is consistent with Penn Aa, Ab and Abc.

**Table:** 

**Authors' roles & responsibilities**	
FR	Substantial contributions to the conception or design of the work; or the acquisition, analysis or interpretation of data for the work; final approval of the version to be published
DP	Substantial contributions to the conception or design of the work; or the acquisition, analysis or interpretation of data for the work; final approval of the version to be published
GS	Substantial contributions to the conception or design of the work; or the acquisition, analysis or interpretation of data for the work; final approval of the version to be published
RR	Substantial contributions to the conception or design of the work; or the acquisition, analysis or interpretation of data for the work; final approval of the version to be published
VD	Substantial contributions to the conception or design of the work; or the acquisition, analysis or interpretation of data for the work; final approval of the version to be published
